# *Operando* structural study of non-aqueous Li–air batteries using synchrotron-based X-ray diffraction†

**DOI:** 10.1039/c8ra04855j

**Published:** 2018-07-23

**Authors:** Chulho Song, Kimihiko Ito, Osami Sakata, Yoshimi Kubo

**Affiliations:** Global Research Center for Environment and Energy based on Nanomaterials Science (GREEN), National Institute for Materials Science (NIMS) 1-1 Namiki Tsukuba Ibaraki 305-0044 Japan KUBO.Yoshimi@nims.go.jp +81-029-860-4773; Synchrotron X-ray Station at SPring-8, Research Network and Facility Services Division, National Institute for Materials Science (NIMS) 1-1-1 Kouto, Sayo Hyogo 679-5148 Japan

## Abstract

Non-aqueous lithium–air batteries (LABs) attract attention as a candidate technology for next-generation energy storage devices. It is crucial to understand how the discharge product Li_2_O_2_ is formed and decomposed by the electrochemical reactions to improve the cycle performance and decrease the charge voltage, which are the most important subjects for LAB development. Here, *operando* X-ray diffraction with high-brilliant X-rays in a transmission mode was used to observe the intensity and structural changes of crystalline Li_2_O_2_ in an operating non-aqueous LAB in real time, and the Li–O_2_ electrochemical reaction involving Li_2_O_2_ formation and decomposition was clearly demonstrated. The electrochemically formed Li_2_O_2_, which had an anisotropic domain size of 10 nm in the *c*-direction and 40–70 nm in the *ab*-plane, grew due to the increase of the number of domains during the discharge process. No other reaction products with a crystalline phase such as LiOH were found in either the cathode or anode of the LAB, whereas the accelerated decomposition rate of the domains was accompanied with the change of the domain shape and lattice constant of the *c*-axis in the latter half of the charge process with voltage higher than 4 V.

## Abstract 2

The Li–O_2_ electrochemical reaction involving the formation and decomposition of crystalline Li_2_O_2_ was clearly demonstrated by using an *operando* synchrotron-based XRD in a transmission mode and a special airtight LAB cell.

## Graphical abstract

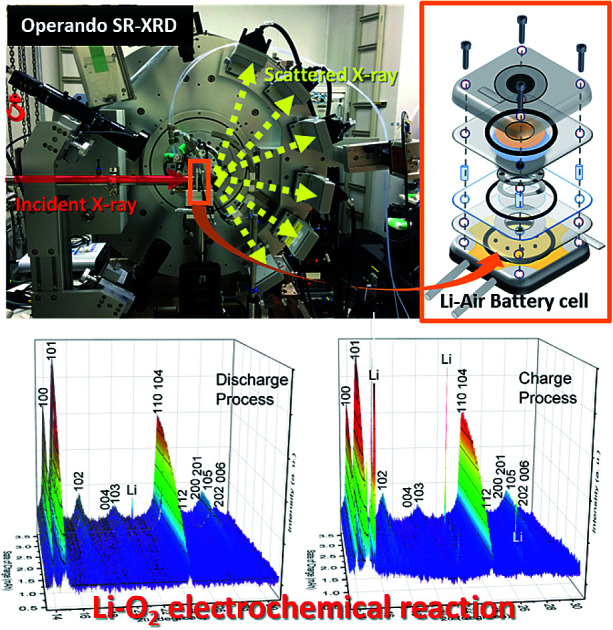

## 1 Introduction

Rechargeable non-aqueous lithium–air batteries (LABs) are attractive as next-generation energy storage devices to enable the shift from fossil fuels to renewable energy sources because of their high theoretical specific energy and specific capacity.^1–6^ However, non-aqueous LAB technology is still in its infancy regarding commercialisation for use as energy storage devices. Current drawbacks such as poor cycle ability^7^ and a large overpotential in the charge process^8^ need to be overcome for a commercial application of LABs. To improve the cycle performance and lower the charge voltage of LABs, it is important to elucidate the Li–O_2_ electrochemical reaction that has the following equation:

1 2Li^+^ + O_2_ + 2e^−^ ↔ Li_2_O_2_ (s), *E*^0^ = 2.96 V *vs.* Li/Li^+^

Namely, for discharge/charge processes of non-aqueous LABs, the formation and decomposition of Li_2_O_2_ as a reaction product need to be thoroughly understood. Understanding of side reactions, particularly LiOH formation, during the operating process of LABs is also necessary.

A general technique used to identify the existence of crystalline Li_2_O_2_ in discharged cathodes is conventional^9,10^/*ex situ* X-ray diffraction (XRD).^4,11,12^ Previous studies using conventional/*ex situ* XRD have involved the removal of the cathode from the LAB cell after full discharge and then collecting XRD data for the cathode using a laboratory-based XRD instrument with a low signal-to-noise (S/N) ratio. Thus, information of the growth process of crystalline Li_2_O_2_ could not be obtained especially both in the initial stage of discharge and the final stage of recharge processes, where the amount of discharge product of Li_2_O_2_ is small. In addition, there is an unavoidable risk that Li_2_O_2_ qualitatively changes by extracting the cathode from a test cell (*e.g.* formation of LiOH).

*In situ* or *operando* XRD is a powerful technique to monitor the structural changes of reaction products with a crystalline phase in LAB cells during operation. To investigate the reaction products of LABs, it is very important to design a LAB cell for measurement purposes that minimises unintended exposure to the ambient environment during an operation. A laboratory-based *in situ* XRD technique in reflection mode has been used to study the Li–O_2_ electrochemical reaction in specially designed LAB cells.^13,14^ However, this *in situ* XRD technique used an X-ray source of low-brilliance (Cu K_α_), so it required a long scan time of 30–70 min to obtain XRD patterns including only three diffraction peaks. This long scan time is inconvenient for studying the Li–O_2_ electrochemical reaction. A synchrotron-based *in situ* X-ray technique^15–20^ has been used to collect high-quality XRD patterns in a short scan time. Storm *et al.*^15^ designed a capillary-based Li–O_2_ battery consisting of an electrolyte-filled capillary with the anode and cathode on opposite ends coated on stainless steel wires. Shui and colleagues^16,17^ used a microfocused synchrotron XRD technique to investigate the reversibility of anodic lithium, and Li_2_O_2_ grain growth and its distribution inside a Swagelok-type cell. They observed the conversion of metallic Li to LiOH on the anode in their investigation of the reversibility of anodic lithium formation.^16^ This phenomenon is a cause of cycle performance degradation in LABs. The conversion of metallic Li to LiOH may originate from the following two reasons: (1) reaction of Li with water produced from electrolyte decomposition; (2) issues controlling the ambient environment of the Li–O_2_ battery cell. It is necessary to confirm which of these reasons results in LiOH formation. The information derived from these studies is of value to understand the electrochemical reactions that occur in LABs.

In this study, we aim to clarify the relationship between the reversible electrochemical reaction and structure of crystalline Li_2_O_2_ and examine the LiOH formation on both a cathode and an anode in a non-aqueous LAB during an operation. For this purpose, we have to strictly eliminate the influence from an ambient water and detect structural information about reaction products in a LAB cell in a very short time without stopping discharge and charge operation. We made a special airtight cell and employed synchrotron-based XRD in a transmission mode with multi-channel detectors to detect simultaneously diffracted X-rays all over angular range of interest. Those enable us to measure high quality XRD data and carry out Rietveld refinement analysis which finally lead to attain real-time structural evolution of crystalline Li_2_O_2_ in an operating LAB cell.

## 2 Experimental

### 2.1 Sample preparation

#### 2.1.1 Cathode preparation

The cathode was constructed by mixing porous carbon (CNovel, pore size: 5 nm, Toyo Tanso Co., Ltd.) and polyvinylidene fluoride (PVDF) binder (10 wt% of the porous carbon) to form a slurry. The slurry was coated onto a gas diffusion layer (carbon paper) to give a carbon loading of 1.0 mg_carbon_ cm^−2^.

#### 2.1.2 LAB cell assembly

A LAB cell was specially designed for *operando* synchrotron-based XRD measurements. The cell was assembled in an Ar-filled glove box by sandwiching an as-prepared cathode (diameter: 16 mm), porous separator (glass microfiber, thickness: 0.26 mm, Whatman) and Li foil. The window of the LAB cell was Al-coated Kapton. The details and schematic drawing of the Li–air battery cell assembly is described in Section 1 of the ESI.† The area where electrochemical reaction proceeds was 1 cm^2^. The electrolyte was 1 M LiCF_3_SO_3_/tetraethylene glycol dimethyl ether (TEGDME).

### 2.2 Synchrotron-based XRD in a transmission mode

*Operando* synchrotron-based XRD experiments in a transmission mode were performed at beamline BL15XU (SPring-8, Japan) using an X-ray of energy 19 keV (*λ* = 0.653 Å). The experimental set-up used for *operando* synchrotron-based XRD measurements is shown in Fig. S2 of the ESI.† Diffraction patterns were collected at angle intervals of 0.01° using six one-dimensional detectors. Each XRD profile was collected in the 2*θ* region of 1.63–74.37° every 5 min. The exposure time was 22 s. This measurement technique has several advantages for LAB investigation. The high-energy X-rays can penetrate the entire LAB cell without disturbing cell operation; therefore, one can observe phase and structural changes simultaneously as they occur. The exposure time of 22 s is much shorter than the operating duration of the LAB cell, making it feasible to observe the formation/decomposition of crystalline Li_2_O_2_ at the cathode region in real time.

## 3 Results and discussion

### 3.1 Discharge/charge voltage profiles

**Fig. 1:**
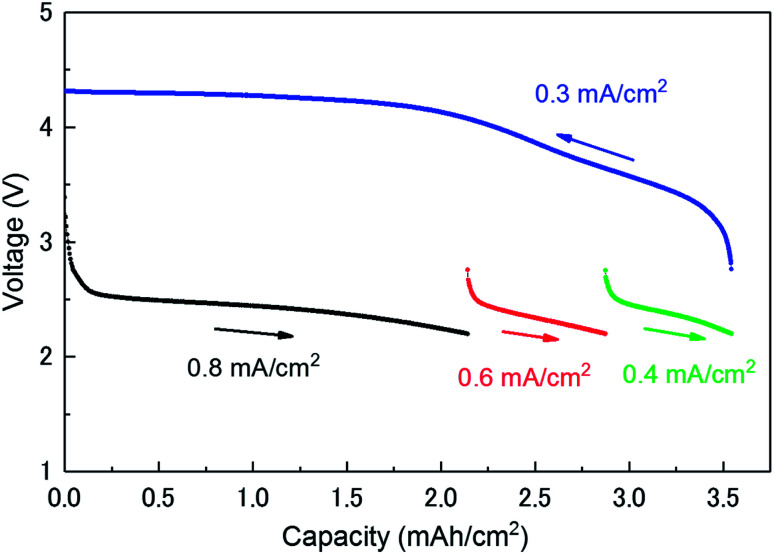
Discharge/charge voltage profiles of the investigated lithium–air battery.

Fig. 1 shows the discharge/charge profiles of the investigated LAB. The discharge processes were limited at a cut-off voltage of 2.2 V. The discharge processes were carried out at three different current densities (0.8, 0.6, and 0.4 mA cm^−2^) until a capacity of 3.54 mA h cm^−2^ was reached. The charge process was carried out at a current density of 0.3 mA cm^−2^ from 3.54 to 0 mA h cm^−2^. The charging potential curve is in agreement with that reported in the literature.^13,17^ As it can be seen in Fig. 1, the voltage steeply decreases just after the beginning of discharge until the amount of the capacity reaches at ∼0.15 mA h cm^−2^. The current of the region mainly originates from the discharge in an electrical double-layer between a carbon and an electrolyte in a cathode (double-layer capacitance). Its capacity can be roughly estimated to be 25 mF from the slope of a discharge profile. This value can be easily verified from the specifications of the cathode. The specific surface is about 0.8 m^2^ mg^−1^ (800 m^2^ g^−1^ for CNovel carbon). The capacity of the double layer at the interface between the carbon and the electrolyte is expected to be 28 mF when relative permittivity of electrolyte and the thickness of the double layer is assumed to be 4 and 1 nm, respectively.

### 3.2 *Operando* XRD patterns

**Fig. 2:**
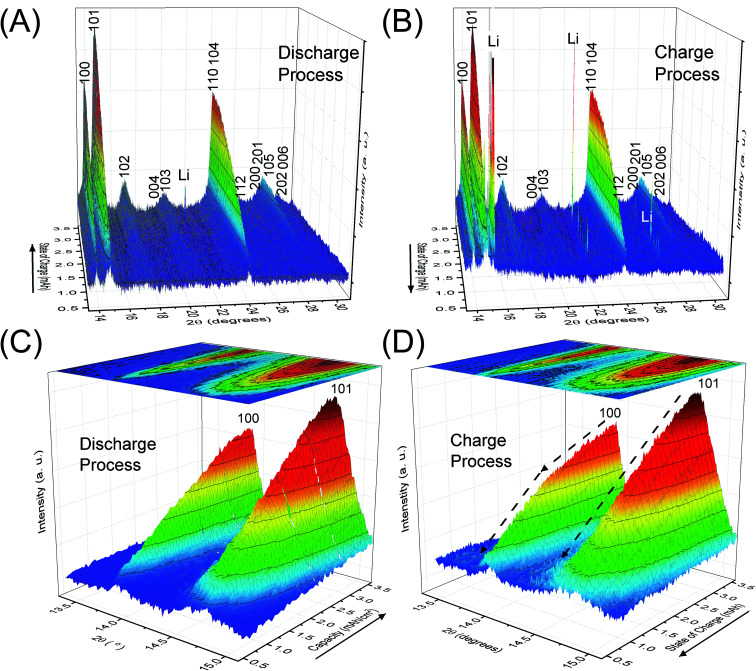
*Operando* XRD patterns of crystalline Li_2_O_2_ in the lithium–air battery cell during (A) and (C) the discharge and (B) and (D) the charge processes. The direction of the solid arrows represents the flow of time.

Fig. 2 depicts the change of XRD patterns for the reaction products in the LAB cell collected during the discharge/charge processes illustrated in Fig. 1. Fig. 2A and B show time-dependent XRD patterns from 2*θ* = 13.3 to 31.0° for the discharge and charge process, respectively. Fig. 2C and D are the enlarged XRD patterns of 2*θ* = 13.3 to 15.1° for Fig. 2A and B, respectively. Miller indices, shown for number of peaks in Fig. 2, indicate the reflections of crystalline Li_2_O_2_. The direction of the solid arrows, shown next to the capacity axes in Fig. 2, represents the increase of operation time of the LAB cell. The XRD patterns were obtained by subtracting the XRD profile of the fresh LAB cell from each XRD profile of the discharged LAB cells. Details of XRD pattern collection are presented in Section 2 of the ESI.† Notably, regardless of a very short scanning time of 22 s, the S/N ratio of the obtained XRD patterns were better than that of the previous study^14^ of a laboratory-based *in situ* XRD. These high-quality XRD patterns enable us to conduct more accurate structural analysis during the operating process of LABs as precisely described after the next section.

### 3.3 Rietveld refinement

**Fig. 3:**
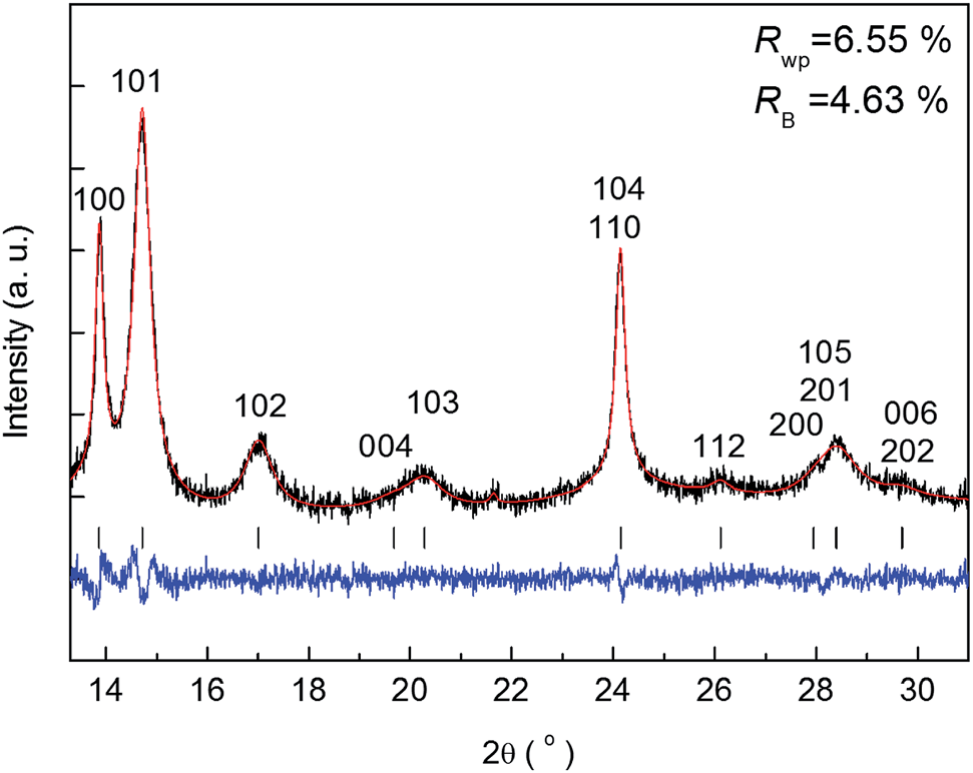
Rietveld refinement of a crystalline Li_2_O_2_ XRD pattern after full discharge (3.54 mA h cm^−2^) of the lithium–air battery. The black, red, and blue line represent the measured pattern (*I*_obs_), the calculated pattern (*I*_cal_), and the difference (*I*_obs_ − *I*_cal_), respectively.

The benefit of the XRD data with a high S/N ratio superior to the previous reports,^13,14^ as shown in Fig. 2, enables us to perform the Rietveld refinement analysis using the pseudo-Voigt function with a reflection asymmetry to precisely elucidate the structural changes of crystalline Li_2_O_2_ in an operating LAB. The results of the Rietveld refinement analysis for a discharge product Li_2_O_2_ are presented in Fig. S4 of the ESI.† The Bragg *R* factor (*R*_B_), which shows the accuracy of the refinement procedure, is also presented in Fig. S5 of the ESI.† As a typical example, the Rietveld refinement of the Li_2_O_2_ XRD pattern after full discharge (3.54 mA h cm^−2^) of the LAB is presented in Fig. 3, and the obtained *R*_B_ is kept less than 5%. Such good *R*_B_s are obtained for the cathode discharged more than 1 mA h, and even for the case of residual charge of less than 1 mA h, *R*_B_s are usually kept less than 20%. The *R*_B_s of residual components are small enough to discuss the structural change in the discharge product as shown in Fig. S4 of the ESI.†*R*_B_s larger than 20% sometimes elucidated are due to appearance of diffraction line from polycrystalline Li metal stronger than that of Li_2_O_2_, which tends to appear near the end region of the charging process. It is noted that peaks corresponding to the Li anode occasionally appear at 15.2°, 21.5°, and 26.5° as indicated by “Li” in Fig. 2, which frequently appear during the charge process than during the discharge process. This result indicates the expected anode reaction in LAB where Li ions are electrochemically deposited on the surface of Li anode in a polycrystalline form with small grain size, thus X-rays diffracted from those are often detected during the charging process. On the other hand, grain size of the original Li foil used in our experiments is so large that its diffraction pattern is spotty similar as a single crystal material. Such diffracted X-rays are rarely detected in our experimental setup at the initial and during the discharge process. It is noted again that: (1) no XRD peaks from other reaction products such as Li_2_O, LiOH, and Li_2_CO_3_ with a crystalline phase were found at the cathode or anode during the operation, although these results do not exclude the possible existence of non-crystalline products due to side reactions in the LAB system during the operation. Shui *et al.*^17^ observed the main reaction product in the cathode is Li_2_O_2_. While they reported the conversion of metallic Li to LiOH on the anode,^16^ we hardly observed crystalline LiOH. This discrepancy may be due to differences in the components such as electrolyte, and/or the difference in airtightness of the *operando* cell. (2) The XRD patterns for Li_2_O_2_ could be refined using the *P*6_3_/*mmc* space group. In addition to the phase identification, some important structural parameters were calculated from the results of Rietveld analysis, such as formation/decomposition rate, anisotropic domain size, and lattice parameters. The full width at half maximum (FWHM) of XRD peaks in the *c*-direction is anisotropically broadened, unlike that of commercial bulk Li_2_O_2_. Details of the FWHM and relative intensity ratio of the XRD peaks of commercial bulk Li_2_O_2_ and discharge product Li_2_O_2_ are described in Section 3 of the ESI.† The details of analysis results for the series of XRD patterns are given in the following sections. From the such control of the experimental environments, which leads an accurate Rietveld analysis, we can discuss the truly real-time (*operando*) appearance and disappearance of peaks corresponding to the reaction product of crystalline Li_2_O_2_ in Fig. 2 and confirm that the electrochemical reaction proceeds dominantly according to eqn (1). In more detail in the evolution of Li_2_O_2_ as found in Fig. 2C and D, the change of peak intensity corresponding to the 100 reflection for the discharge process was clearly different to that for the charge process; the peak intensity of the 100 reflection increases linearly on discharge (Fig. 2C) whereas it decreases nonlinearly with two steps during charging (Fig. 2D). Similar decrease in peak intensities during the charge process was observed in the previous *in situ* XRD study,^14^ which reported that the peak intensities of the 100 and 101 reflections decrease nonlinearly with two steps. As it can be seen in Fig. S7 and Table S2 of the ESI,† the relative intensity ratio for 100 peaks hardly change and the FWHM for 100 peaks decrease during the charge process. The nonlinearity in the charge process implies that the decomposition of crystalline Li_2_O_2_ involves two stages and structural changes are greatly influenced by crystallographic direction during the charge process. Here, it should be noted that the 2^nd^ step starts from around 2.28 mA h cm^−2^ corresponding to the charge voltage of 4 V.

### 3.4 Variation of the integrated and normalised areas of crystalline Li_2_O_2_ peaks

**Fig. 4:**
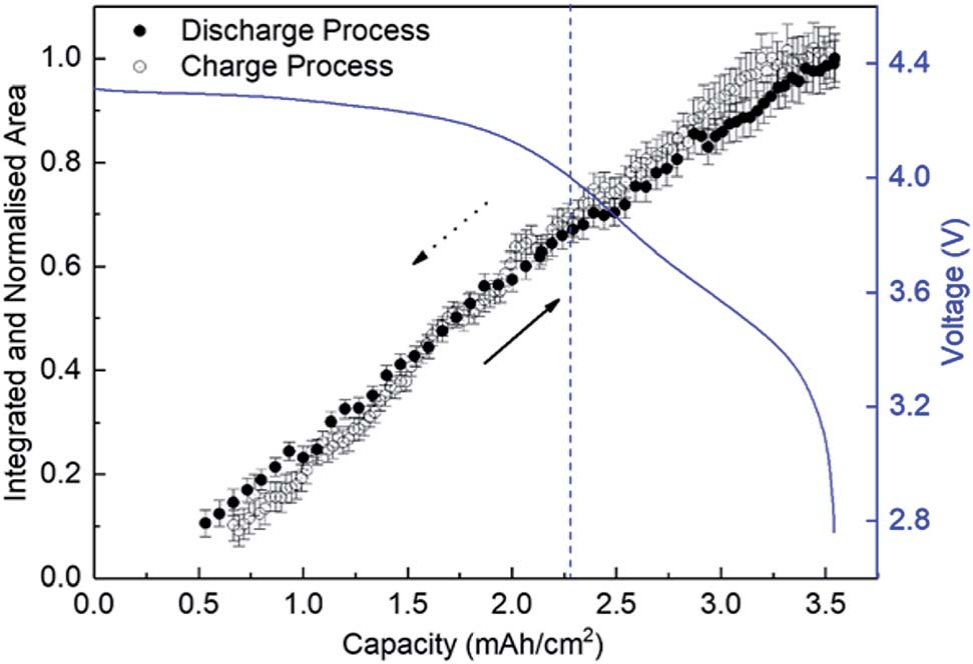
Integrated and normalised areas of crystalline Li_2_O_2_ peaks during the discharge and charge processes. The line-arrows and dotted-arrows in a black colour show the directions of discharge and charge processes, respectively.

Fig. 4 shows the variation of the integrated and normalised areas of crystalline Li_2_O_2_ peaks in the 2*θ* range of 13.3–31° during the discharge/charge processes. Here, the integrated area was calculated by summing all areas of 13 Li_2_O_2_ peaks in the results of the Rietveld analysis of each XRD pattern, which were normalised by the integrated area of a fully discharged LAB (3.54 mA h cm^−2^). The error bars in Fig. 4 were presented by using *R*_B_. For a discharge process, the integrated and normalised areas of Li_2_O_2_ peaks increase linearly, without a change of slope, irrespective of current density (0.8, 0.6, and 0.4 mA cm^−2^ in Fig. 1). The extrapolated curves for the discharge process do not cross the origin (capacity = 0.114 mA h cm^−2^ for integrated and normalised areas = 0) (see Fig. S8 of the ESI†). This amount of charge is almost the same as 0.15 mA h cm^−2^ and is interpreted by discharge from the double-layer explained previously, thus it is reasonable that Li_2_O_2_ does not grow in the range from 0 to 0.114 mA h cm^−2^ because the electrical current in this region does not have electrochemical contribution (namely, Faraday current). It is also hard to conclude that Li_2_O_2_ exists in an amorphous state until 0.114 mA h cm^−2^, or some electrons from the Li anode in the LAB cell participate in a reaction other than Li_2_O_2_ formation. For the charge process, at first glance overall, the integrated and normalised areas of Li_2_O_2_ peaks also decrease linearly, which indicates that the reversible Li–O_2_ electrochemical reaction involving crystalline Li_2_O_2_ formation/decomposition occurs. A plateau region at the beginning of the charge process between 3.54 and 3.25 mA h cm^−2^, showing little change of integrated and normalised areas, can be explained again by charging to the double-layer as mentioned above. In more detail, the decreases in the integrated and normalised areas of Li_2_O_2_ peaks seem to accelerate slightly from the latter half of the charge process (below 2.28 mA h cm^−2^). As seen in the intensity change of 100 reflection shown in Fig. 2, it is confirmed again that the manner of electrochemical reaction switched to a somewhat different one after 2.28 mA h cm^−2^ during the charging process.

### 3.5 Variation of the domain size of crystalline Li_2_O_2_

**Fig. 5:**
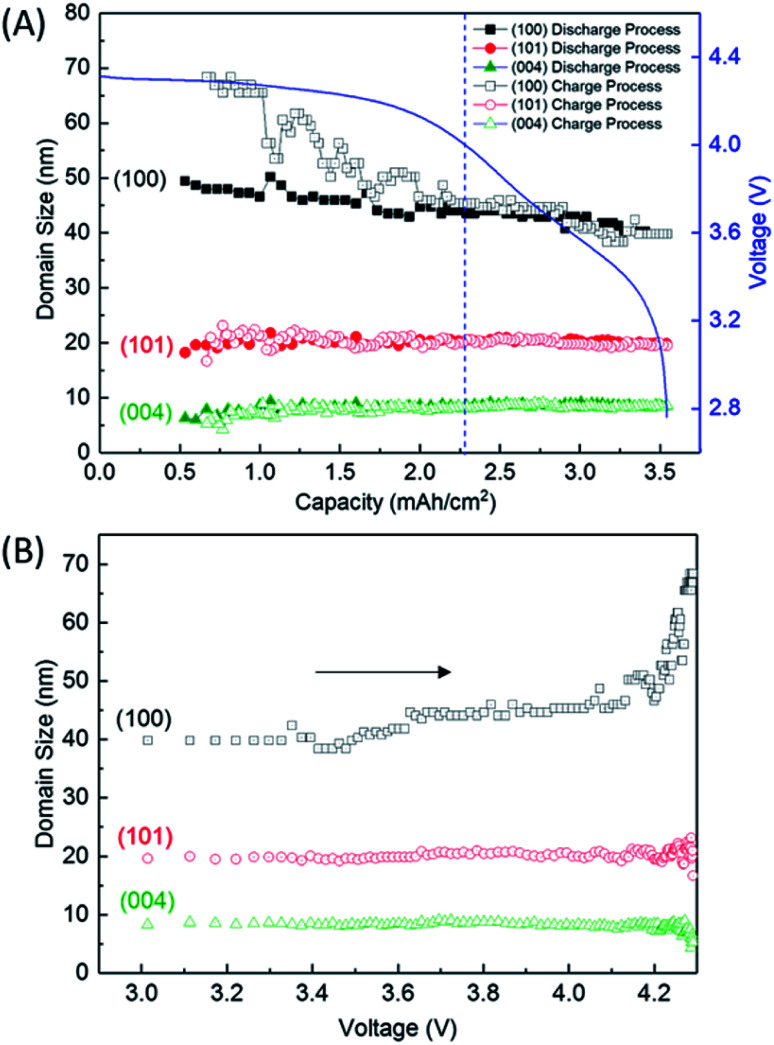
Domain sizes of crystalline Li_2_O_2_ (A) as a function of capacity during the discharge and charge processes and (B) as a function of voltage during the charge process, which was replotted by converting amount of charge in (A) to voltage using the charging profile. The direction of the arrows in (B) represents the flow of charge process.

Fig. 5A shows the variation of domain size of crystalline Li_2_O_2_ calculated from the FWHM of the 100, 101, and 004 peaks during the discharge/charge processes. The FWHM values are presented in Table S2 of the ESI.† The notable difference of FWHM values of 100, 101, and 004 peaks indicates that the crystalline Li_2_O_2_ possesses anisotropic morphology. The domain size was calculated from the Scherrer equation:

2 *D* = *Kλ*/*β* cos *θ*

where *D* is a domain size, *K* is the shape factor, *λ* is the X-ray wavelength, *β* is the line broadening at the FWHM of the observed peak in radians, and *θ* is the Bragg angle. To obtain precise *D*, we used a value of *β* that was corrected by subtracting the effect of instrumental broadening from the experimentally observed peaks (*β*^2^_real_ = *β*^2^_observed_ − *β*^2^_instrumental_). The magnitude of instrumental broadening (*β*^2^_instrumental_) was estimated using a standard powder (CeO_2_, NIST SRM 674a, 200 nm), as illustrated in Fig. S9 of the ESI.† The calculated domain sizes of crystalline Li_2_O_2_ in the fully discharged LAB (Fig. 3) were 39.8, 19.5, and 8.6 nm for the 100, 101, and 004 reflections, respectively. Such anisotropic domain shape agrees with that reported in the literature.^14,15^ It is notable in our study that the domain size of the crystalline Li_2_O_2_ hardly changes without a change of slope irrespective of current density (0.4, 0.6, and 0.8 mA cm^−2^), during the discharge process (Fig. 5A). This result directly means that the growth of crystalline Li_2_O_2_ mainly involves an increase of the number of crystalline Li_2_O_2_ particles with small crystallographic domains, which was not reported in previous *in situ* XRD studies.^13,17^ In ref. 13, it is reported that domain size, estimated from 101 reflection, decreases from 40 to 25 nm with increase of discharge capacity. On the other hand, in ref. 17, it is reported the domain size increase from 4 to 9.3 nm. Those discrepancies about the variation of the domain size may come from the difference in the specification of a cathode and an electrolyte, especially the water content in electrolyte. The water content in the cell was verified low enough from the domain size calculated above, which has no discrepancy to the reported data.^21^ Furthermore, the domain size for the 100 reflection tends to increase, in addition to the change of decreasing rate of peak intensity for the 100 reflection shown in Fig. 2D, in the latter half of the charge process (below 2.28 mA h cm^−2^). This does not necessarily mean that the particle size has increased. A possible interpretation is that smaller particles decomposed first and bigger particles remained. It is also found that the domain size for the 100 reflection is very sensitive to the change of voltage, shown in Fig. 5B. This phenomenon is in agreement with a previous study.^14^ However, this tendency is not conclusive because of the large error (see Fig. S5 of the ESI†) for Rietveld refinement analysis that originates from the weak XRD intensity for the crystalline Li_2_O_2_ in this charge region. At least, the minimum signal to S/N ratio seems to be secured for the 100 reflection.

We finally discussed the variation of lattice constants of crystalline Li_2_O_2_ during the operation in Section 7 of the ESI.† The lattice constant of the *a*-axis has no variation during the discharge process. However, the lattice constant of the *c*-axis looks to increase gradually from 2.28 mA h cm^−2^ during the charge process. In addition to this result, the increase of domain size for the 100 reflection in Fig. 5 seems to be accompanied with the accelerated electrochemical decomposition of crystalline Li_2_O_2_ above 4 V for the charge process, as shown in Fig. 4. It is hard to explain such changes especially in domain size and lattice constant during the charging process under the assumption of the main electrochemical reaction described by eqn (1). It is natural to think that the path of electrochemical reaction involving Li_2_O_2_ switches to different one when the voltage exceeds the threshold of the electrochemically stable window of the electrolyte or carbon. In this case, 4 V might be the criterion voltage. As a result, the decomposition rate and the shape of domain of Li_2_O_2_ are affected. As a supporting evidence, the drastic evolution of CO_2_, instead of O_2_, was actually observed at voltage higher than 4 V during a charge process for a TEGDME-based Li–air battery cell, as shown in Fig. S11 of the ESI.† This result indicates the occurrence of electrochemical decomposition of a solvent or carbon cathode itself. Advanced analysing technique, which enables us to understand microscopically the chemical state of lithium, are needed to elucidate more precisely the detail of this electrochemical reaction.

Finally, it should be emphasized from the technical point of view that such small changes in the structural parameters of crystalline Li_2_O_2_ in a LAB cell in operation can be observed in real time using our *operando* XRD measurement system.

## 4 Conclusions

To elucidate the structural changes of crystalline Li_2_O_2_ and examine the LiOH formation in an operating LAB, *operando* synchrotron radiation-based XRD experiments in a transmission mode were conducted. The Li–O_2_ electrochemical reaction involving the formation and decomposition of crystalline Li_2_O_2_ was clearly demonstrated. Rietveld refinement analysis revealed that the domain size of anisotropic crystalline Li_2_O_2_ platelets, which were 10 nm in the *c*-direction and 40–70 nm in the *ab*-plane, was not influenced by the current density, and the growth of disc- and toroid-shaped Li_2_O_2_ particles was not accompanied with the growth of crystalline domains during the discharge process. No other reaction products with a crystalline phase as LiOH were observed in either the cathode or anode of LAB during the operation, whereas the accelerated electrochemical decomposition of the crystalline Li_2_O_2_ was accompanied with the increase of the lattice constant of the *c*-axis/the domain size for the 100 reflection and the drastic evolution of CO_2_ in voltage higher than 4 V of the charge process. This information about the structural changes within a non-aqueous LAB during an operation furthers our understanding of the nature of the Li–O_2_ electrochemical reaction in non-aqueous LABs, and should aid fabrication of high-performance non-aqueous LABs.

## Conflicts of interest

There are no conflicts to declare.

## Supplementary Material

RA-008-C8RA04855J-s001
