# Supplementary material for: Operando structural study of non-aqueous Li–air batteries using synchrotron-based X-ray diffraction
Source: RSC Adv. 2018 Jul 23;8(46):26293–9. doi: 10.1039/c8ra04855j (PMC9082782; doi:10.1039/c8ra04855j)
Supplement: RA-008-C8RA04855J-s001 [file RA-008-C8RA04855J-s001.pdf]

Electronic Supplementary Material (ESI) for RSC Advances.
This journal is © The Royal Society of Chemistry 2018

Electronic Supplementary Information

Operando structural study of non-aqueous Li–air batteries using synchrotron-based X-ray diffraction

Chulho Song,[ab] Kimihiko Ito,[a] Osami Sakata,[ab] and Yoshimi Kubo*[a]

[a] Global Research Center for Environment and Energy based on Nanomaterials Science (GREEN), National Institute for Materials Science (NIMS), 1-1 Namiki, Tsukuba, Ibaraki 305-0044, Japan

[b] Synchrotron X-ray Station at SPring-8, Research Network and Facility Services Division, National Institute for Materials Science (NIMS), 1-1-1 Kouto, Sayo, Hyogo 679-5148, Japan

*Corresponding author: E-mail: KUBO.Yoshimi@nims.go.jp

Section 1. LAB cell assembly and experimental set-up for *operando* SR-XRD measurements

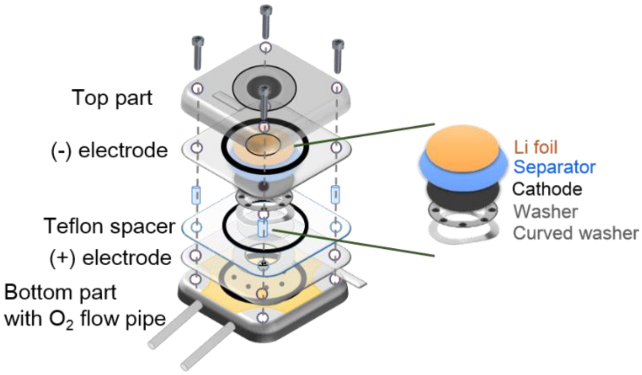

Figure S1. Schematic view of Li-air battery cell assembly.

Figure S1 shows the schematic view of lithium–air battery (LAB) cell assembly. The LAB cell was designed to prevent any unintended exposure to the ambient environment during discharge/charge processes. LAB cell consists of top and bottom parts, (+) (-) electrodes, Teflon spacer, washer, curved washer as well as a conductive porous cathode, an electrolyte, a separator, and a Li foil anode. All gaps between top part, (-), (+) electrodes, and bottom part are sealed with O-rings, that guarantees air tightness. It was confirmed by checking no pressure change for 5 hours or more after filling an Ar gas up to 0.3 MPa in the LAB cell. The windows of top and bottom part were Al-coated Kapton. The bottom part was designed to provide oxygen.

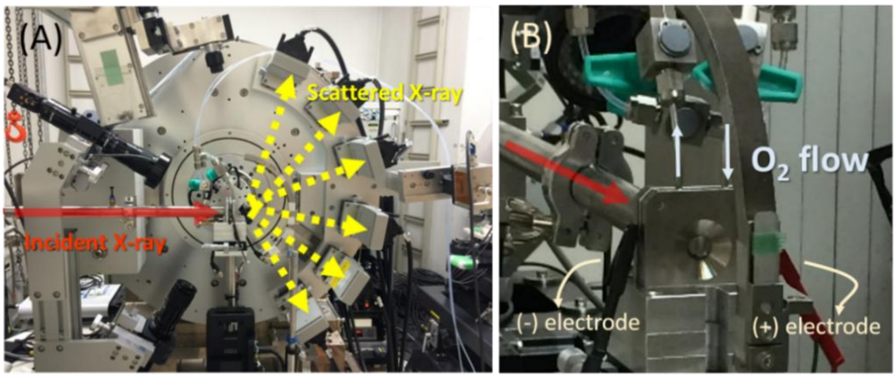

Figure S2. Experimental set-up. (A) Synchrotron-based *operando* XRD study of a lithium–air battery cell under discharge/charge processes. (B) Enlarged view of Fig. S2(A).

Figure S2 shows the experimental set-up used for synchrotron-based *operando* XRD measurements, which was developed to allow time-resolved measurements without the requirements of a scattering angle (2θ) scan. Six one-dimensional detectors were asymmetrically arranged in the perpendicular line as shown in Fig. S2(A). A diffraction intensity profile can be simultaneously recorded in the 2θ angular range from 1.63° to 74.37° in a "one shot" measurement using this experimental set-up. The details of this set-up are described in Ref. S1. The water content in the gap space above the cathode, which was experimentally confirmed by measuring the dew point at the downstream of the LAB cell, was kept below 100 ppm by continuously flowing pure (dried) O_2 gas during the measurement

Section 2. High quality XRD patterns and Rietveld refinement analysis for a discharge product

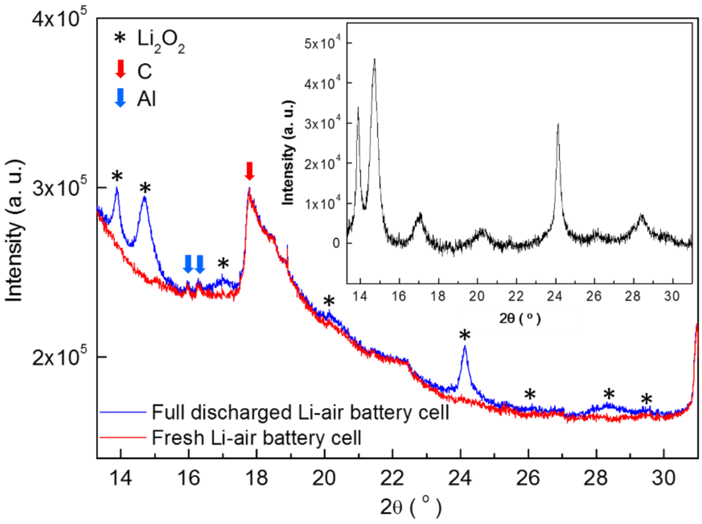

Figure S3. XRD profile for a fully discharged lithium–air battery (LAB) cell (blue line) and fresh LAB cell (red line). The inset is the XRD pattern obtained by subtracting the XRD profile of the fresh LAB cell, before a discharge process, from that of the fully discharged LAB cell.

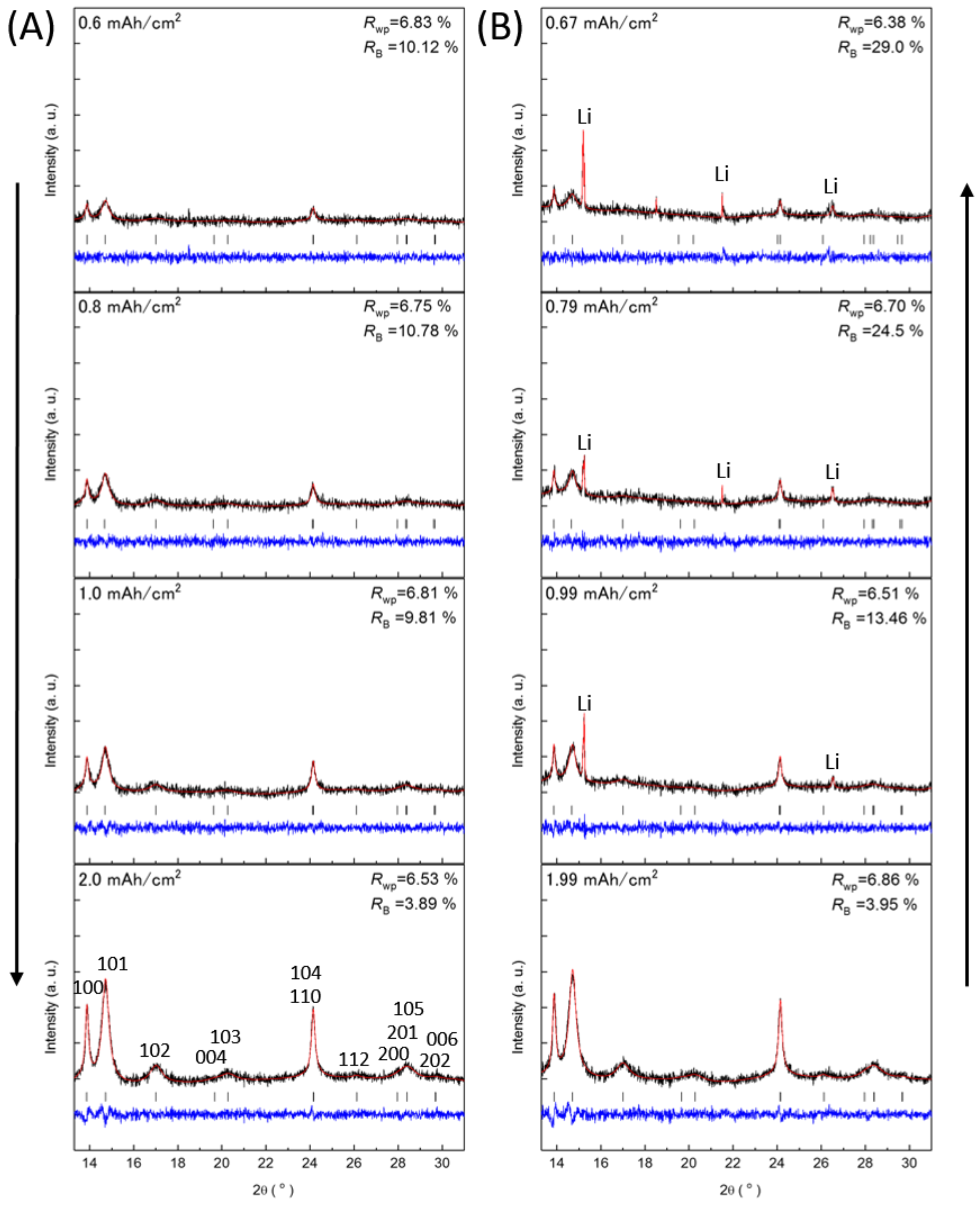

Figure S4. Refinements results for a discharge product Li_2O_2 during (A) discharge and (B) charge processes. The black, red, and blue line represent the measured pattern (I_{obs}), the calculated pattern (I_{cal}), and the difference (I_{obs}-I_{cal}), respectively. The direction of the arrows represents the flow of time.

Figure S3 shows the collection process used to obtain the XRD pattern of discharge products from the XRD profiles for fully discharged and fresh LAB cells. The red line is the XRD profile for the fresh LAB cell before a discharge process, which includes peaks originating from carbon (current collector,

cathode) and aluminium (window material: Al-coated Kapton). The blue line is the XRD profile for a fully discharged LAB cell, which contained only Li_2O_2 peaks in addition to the same ones as the red line. Namely, the XRD patterns of crystalline Li_2O_2 for Rietveld refinement analysis, which is shown in the inset of Fig. S3, can be obtained by subtracting the red line from the blue line.

Figure S4 shows the results of the Rietveld refinement analysis for a discharge product Li_2O_2 during the operating process. The Bragg R factor (R_B), which shows the accuracy of the refinement procedure, is presented in Fig. S5. It is noted that peaks corresponding to the Li anode occasionally appear as indicated by "Li" in Fig. S4(B). In this case, the Rietveld refinement analysis was performed using the pseudo-Voigt (Li_2O_2) and split Pearson-VII (Li) functions. To compare with the crystalline Li_2O_2 XRD pattern after full discharge of the lithium-air battery (3.54 mAh/cm^2) in the Fig. 3, figure S4 was applied on the same scale (x and y axes) of Fig. 3. Notably, it is noted that no XRD peaks from other reaction products such as Li_2O, LiOH, and Li_2CO_3 with a crystalline phase were found during the operating process.

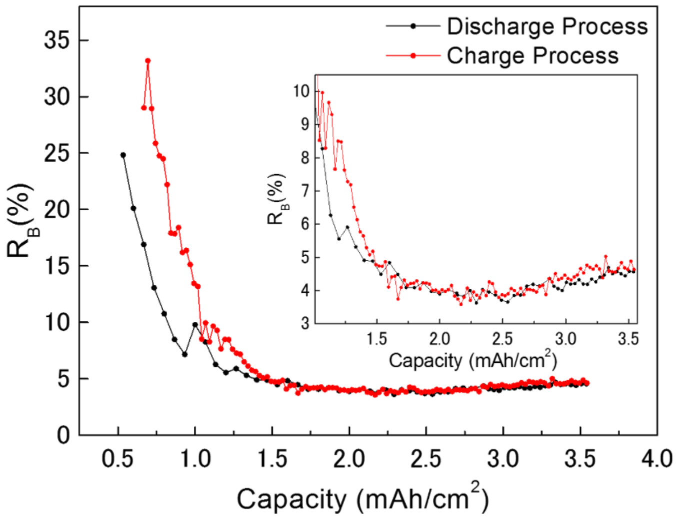

Figure S5. R_B factor for Rietveld refinement analysis on the operating process.

Section 3. FWHM comparison of commercial bulk Li_2O_2 and discharge product Li_2O_2

Figure S6 depicts the XRD patterns of commercial bulk Li_2O_2 and discharge product Li_2O_2 obtained from the LAB cell. Figure S6 reveals that the full width at half maximum (FWHM) of the XRD peaks of the discharge product in the *c*-direction are anisotropically broadened compared with those of commercial bulk Li_2O_2. FWHM and relative intensity ratio values are presented in Table S1.

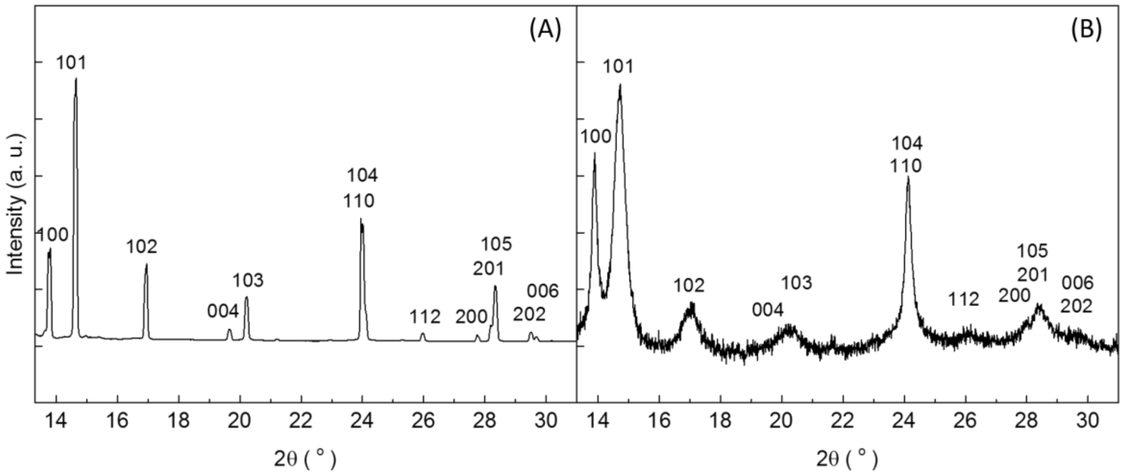

Figure S6. XRD patterns for (A) commercial bulk Li_2O_2, and (B) discharge product Li_2O_2.

Table S1. FWHM and relative intensity ratio of XRD peaks for commercial bulk Li_2O_2 and discharge product Li_2O_2

hkl	FWHM		Integrated intensity ratio (%)	
	Commercial bulk Li_2O_2	Discharge product Li_2O_2	Commercial bulk Li_2O_2	Discharge product Li_2O_2
**100**	0.100	0.086	33	32
**101**	0.099	0.173	100	100
**102**	0.098	0.308	30	34
**004**	0.098	0.393	4	5
**103**	0.099	0.406	16	19
**110**	0.103	0.098	47	33
**104**	0.103	0.461	12	14
**112**	0.106	0.189	4	3
**200**	0.110	0.261	2	2
**201**	0.111	0.467	7	6
**105**	0.112	0.467	27	33
**202**	0.115	0.458	4	5
**006**	0.115	0.458	2	3

Figure S7 shows the variation of relative intensity ratio for 100 and 004 peaks of discharge product Li_2O_2 on the charge process. The relative intensity ratios have no variation during the operation process, except for the beginning of discharge process.

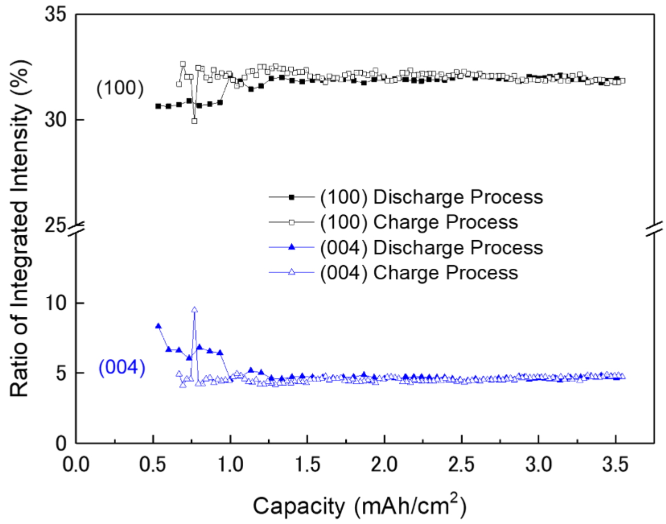

Figure S7. Variation of relative intensity ratios for 100 and 004 peaks on the charge process

Section 4. FWHM and domain size of crystalline Li_2O_2 during LAB operation

Table S2. FWHM and domain size (*D*) of crystalline Li_2O_2 during discharge/charge processes.

Discharge process				Charge process			
mAh/cm^2	Plane	FWHM(2θ)	D (nm)	mAh/cm^2	Plane	FWHM(2θ)	D (nm)
**0.6**	(100)	0.071	48.7	0.67	(100)	0.052	68.4
	(101)	0.172	19.6		(101)	0.202	16.7
	(004)	0.561	6.0		(004)	0.638	5.3
**0.8**	(100)	0.072	48.0	0.79	(100)	0.053	66.9
	(101)	0.170	19.9		(101)	0.157	21.5
	(004)	0.427	7.9		(004)	0.575	5.9
**1.0**	(100)	0.074	46.6	0.99	(100)	0.054	65.5
	(101)	0.159	21.2		(101)	0.158	21.4
	(004)	0.389	8.7		(004)	0.480	7.0
**2.0**	(100)	0.077	44.7	1.99	(100)	0.074	46.6
	(101)	0.164	20.6		(101)	0.168	20.1
	(004)	0.390	8.7		(004)	0.433	7.8
**3.54**	(100)	0.086	39.8	3.54	(100)	0.086	39.8
	(101)	0.173	19.5		(101)	0.173	19.5
	(004)	0.393	8.6		(004)	0.393	8.6

Table S2 lists the FWHM and domain size of crystalline Li_2O_2 during the operation of the LAB cell. The FWHM of the 100 reflections are sharper than those of the 101 and 004 reflections. This result indicates that the crystalline Li_2O_2 has an anisotropic morphology.

Section 5. Integrated and normalised areas of crystalline Li_2O_2 during the discharge process

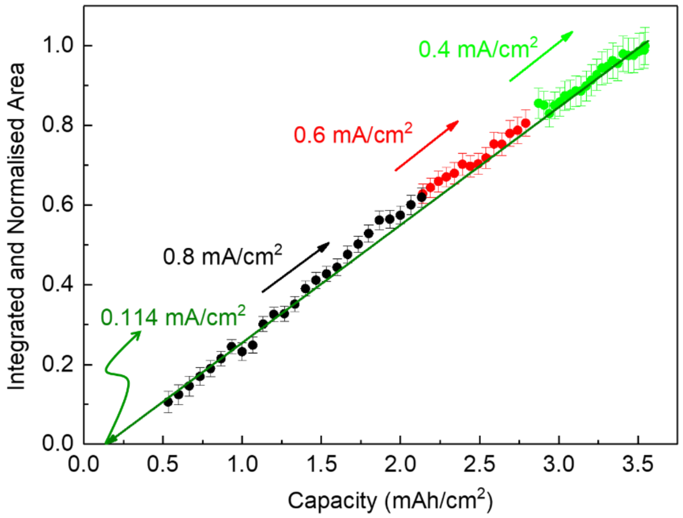

Figure S8. Integrated and normalised areas of crystalline Li_2O_2 peaks during the discharge process.

Figure S8 shows the variation of the integrated and normalised areas of crystalline Li_2O_2 during the discharge process. The integrated and normalised areas of Li_2O_2 peaks increase linearly without a change of slope irrespective of current density (0.4, 0.6, and 0.8 mA/cm^2). From the extrapolation of the curves for the discharge process, the capacity is 0.114 mAh/cm^2 when the integrated and normalised area = 0.

Section 6. Estimation of the magnitude of instrumental broadening

Figure S9 shows the variation of the magnitude of instrumental broadening ($\beta^2_{instrumental}$) as a function of 2θ. In Fig. S5, each point represents the difference between the observed FWHM and theoretical FWHM for a standard powder crystal (CeO_2, NIST SRM 674a, 200 nm). From the fitting results obtained using the equation $(\Delta FWHM)^2 = Utan\theta^2 + Vtan\theta + W,$ the magnitude of instrumental broadening was able to be evaluated.

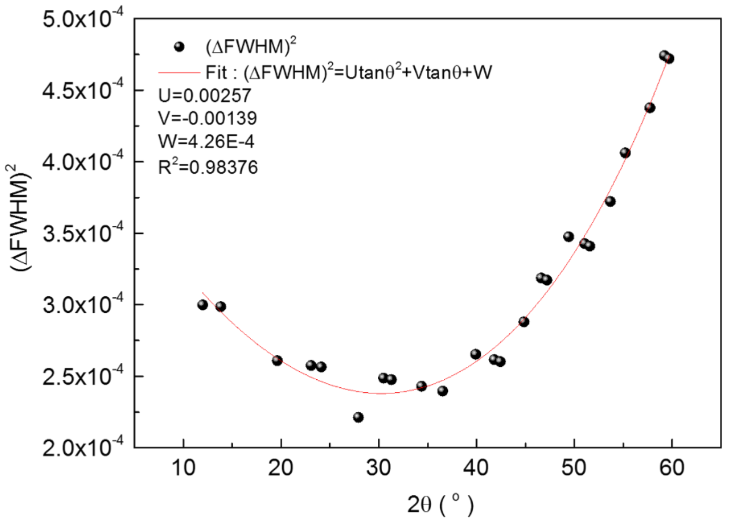

Figure S9. Magnitude of instrumental broadening as a function of 2θ.

Section 7. Lattice constants of crystalline Li_2O_2 during the discharge/charge processes

Figure S10 illustrates the variation of lattice constants of crystalline Li_2O_2 during the operation. The lattice constant of the *a*-axis has no variation during the discharge process. However, the lattice constant of the *c*-axis looks to increase gradually from 2.28 mAh/cm^2 during the charge process. This tendency has not concluded because of the large error (Fig. S5) for Rietveld refinement analysis that originates from the weak XRD intensity for crystalline Li_2O_2 in this charge region.

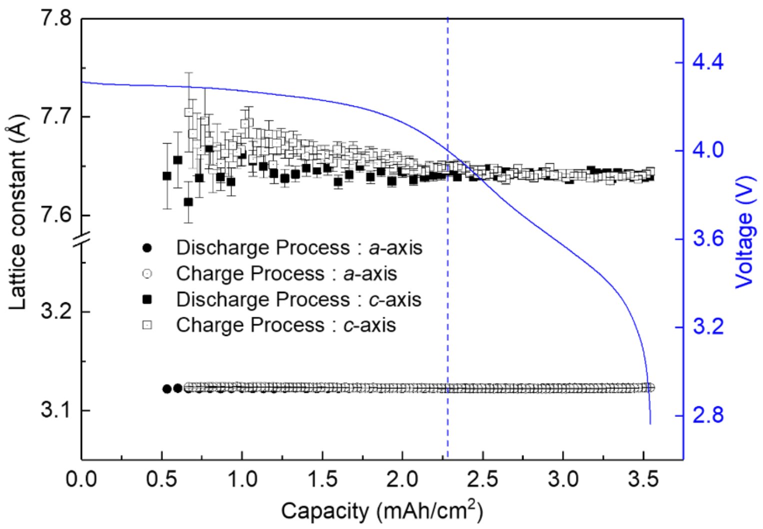

Figure S10. Lattice constants of crystalline Li_2O_2 during the discharge/charge processes.

Section 8. O_2, CO_2 evolution during the charge process

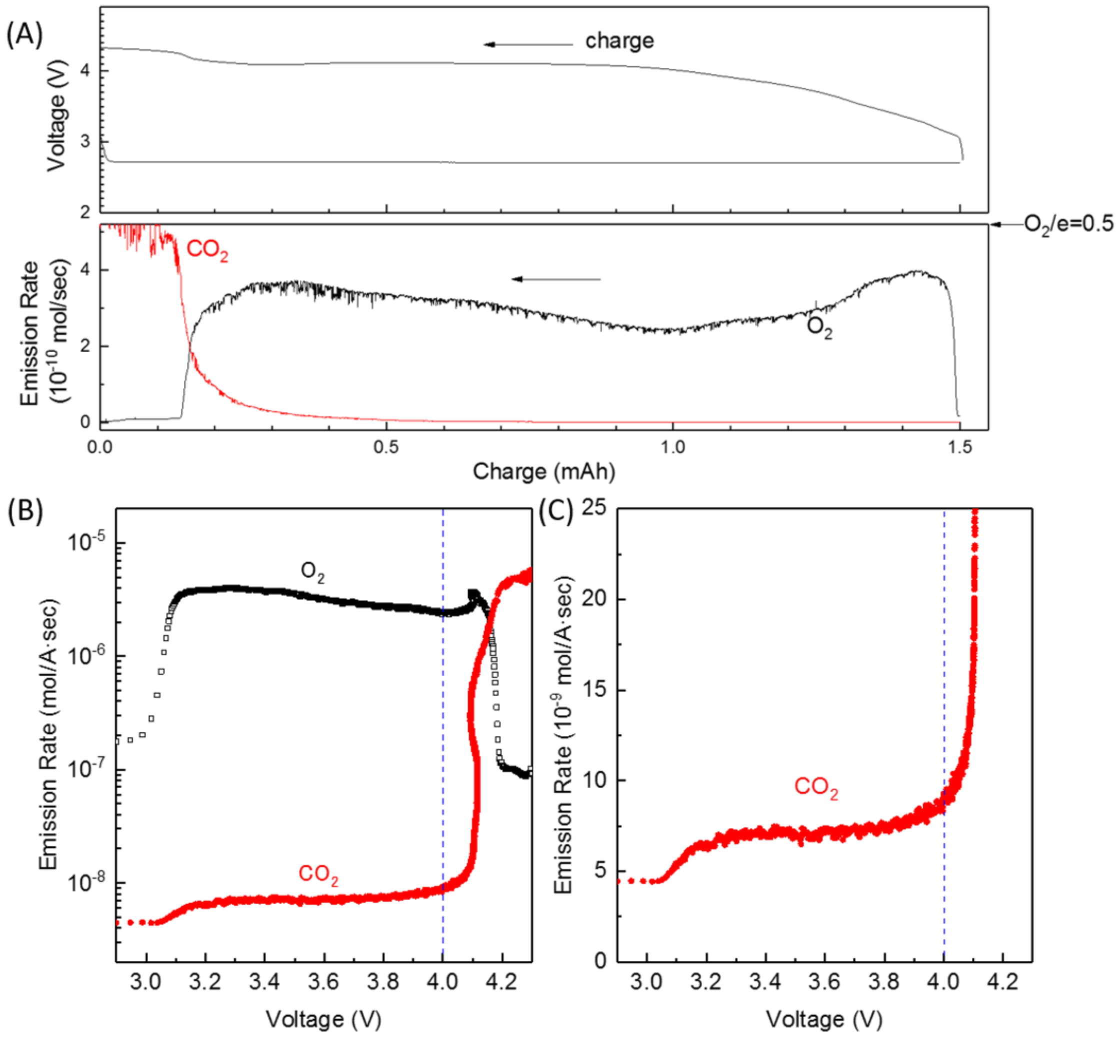

Figure S11. O_2, CO_2 evolution versus (A) cell charge and (B) cell voltage during a charge process for a Li-air battery cell. (C) is the enlargement of CO_2 part in (B).

Figure S11 shows the typical data of differential electrochemical mass spectroscopy during a charge process (CC-mode, the operating current was 100μA) for a tetraethylene glycol dimethyl ether (TEGDME)-based Li-air battery cell. Those data were measured using the same system as that described in Ref. S2. It is found from Fig. S11(A) that O_2 is dominantly exhausted almost in the whole range of charge process except of its end region. Fig. S11(B) is a graph chart replotted by converting amount of charge to voltage using the charge profile shown in Fig. S11(A) and rescaling the emission rate to logarithmic scale. At the beginning and middle of the charge process, the emission rate of CO_2 is in the order of one thousandth of O_2. The CO_2 in this region may be a background, irrelevant to the electrochemical reaction in Li-air battery cell, generated by a reaction of residual carbon and large

amount of oxygen introduced to an ionization chamber of a mass spectrometer. On the other hand, the emission rate of CO_2 drastically increases, in contrast to the decrease of O_2, above ~ 4 V. This fact is very clear when the CO_2 emission rate is replotted in linear scale and enlarged as shown Fig. 11S(C). This phenomenon must be originated from the electrochemical reaction in Li-air battery cell, in which decomposition of the solvent or carbon cathode is thought be involved. And thus, it is natural to think that such change in the way of the electrochemical reaction may be related to the nonlinear decrease with two steps of the peak intensity for the 100 reflection (Fig. 2D) during the charge process, the increase in the domain size for the 100 reflection (Fig. 5) and the lattice constant of the *c*-axis (Fig. S10) in the latter half of the charge process, although the side reaction product such as LiOH is not apparently detected.

Reference

S1. Katsuya, Y. *et al.* An X-ray powder diffractometer with a wide scattering-angle range of 72° using asymmetrically positioned one-dimensional detectors. *Rev. Sci. Instrum.* **87**, 016106 (2016).

S2. Xin, X. *et al.* Highly Efficient Br^-/NO_3^- Dual-Anion Electrolyte for Suppressing Charging Instabilities of $Li-O_2$ Batteries. *ACS Appl. Mater. Interfaces* **9(31)**, 25976-25984 (2017).
